# Evaluation of Protection by Caffeic Acid, Chlorogenic Acid, Quercetin and Tannic Acid against the In Vitro Neurotoxicity and In Vivo Lethality of *Crotalus durissus terrificus* (South American Rattlesnake) Venom

**DOI:** 10.3390/toxins13110801

**Published:** 2021-11-13

**Authors:** Isadora Caruso Fontana Oliveira, Edson Hideaki Yoshida, Murilo Melo Juste Dini, Ana Beatriz Olívio Paschoal, José Carlos Cogo, Maria Alice da Cruz-Höfling, Stephen Hyslop, Yoko Oshima-Franco

**Affiliations:** 1 Post-Graduate Program in Pharmaceutical Sciences, University of Sorocaba (UNISO), Rodovia Raposo Tavares km 92.5, Sorocaba 18023-000, SP, Brazil; isa.cfo@hotmail.com (I.C.F.O.); ehideakiy@gmail.com (E.H.Y.); murilo_juste@hotmail.com (M.M.J.D.); paschoalanabeatriz@gmail.com (A.B.O.P.); 2 Bioengineering and Biomedical Engineering Programs, Technological and Scientific Institute, Brazil University, Rua Carolina Fonseca, 584/235, São Paulo 08230-030, SP, Brazil; jccogo@gmail.com; 3 Department of Structural and Functional Biology, Institute of Biology, State University of Campinas (UNICAMP), Rua Monteiro Lobato, 255, Cidade Universitária Zeferino Vaz, Campinas 13083-862, SP, Brazil; hofling@unicamp.br; 4 Department of Translational Medicine (Section of Pharmacology), Faculty of Medical Sciences, State University of Campinas (UNICAMP), Rua Tessália Vieira de Camargo, 126, Campinas 13083-970, SP, Brazil; hyslop@unicamp.br

**Keywords:** antivenom, phytochemical, rattlesnake venom, tannic acid

## Abstract

Systemic envenomation by *Crotalus durissus terrificus* (South American rattlesnake) can cause coagulopathy, rabdomyolysis, acute kidney injury, and peripheral neuromuscular blockade, the latter resulting in flaccid paralysis. Previous studies have shown that plant products such as tannic acid and theaflavin can protect against the neuromuscular blockade caused by *C. d. terrificus* venom in vitro. In this work, we used mouse-isolated phrenic nerve-diaphragm preparations to examine the ability of caffeic acid, chlorogenic acid, and quercetin to protect against *C. d. terrificus* venom-induced neuromuscular blockade in vitro. In addition, the ability of tannic acid to protect against the systemic effects of severe envenomation was assessed in rats. Preincubation of venom with caffeic acid (0.5 mg/mL), chlorogenic acid (1 mg/mL), or quercetin (0.5 mg/mL) failed to protect against venom (10 μg/mL)-induced neuromuscular blockade. In rats, venom (6 mg kg^−1^, i.p.) caused death in ~8 h, which was prevented by preincubation of venom with tannic acid or the administration of antivenom 2 h post-venom, whereas tannic acid given 2 h post-venom prolonged survival (~18.5 h) but did not prevent death. Tannic acid (in preincubation protocols or given 2 h post-venom) had a variable effect on blood creatinine and urea and blood/urine protein levels and prevented venom-induced leukocytosis. Tannic acid attenuated the histological lesions associated with renal damage in a manner similar to antivenom. The protective effect of tannic acid appeared to be mediated by interaction with venom proteins, as assessed by SDS-PAGE. These findings suggest that tannic acid could be a potentially useful ancillary treatment for envenomation by *C. d. terrificus*.

## 1. Introduction

Envenomation by the South American rattlesnake (*Crotalus durissus terrificus*) is characterized by minimal or no local manifestations, such as pain, mild edema, and recurrent erythema, but marked systemic effects, including coagulopathy, myotoxicity with myoglobinuria, peripheral neuromuscular blockade (flaccid paralysis), and acute kidney injury (AKI) [1,2,3,4,5,6,7,8]. AKI is the principal cause of death [1,4,6], with the interval between envenomation and antivenom administration being an important factor in determining AKI and mortality [6,9]. Antivenom administration is currently the standard treatment for envenomation by *C. d. terrificus* and is effective in attenuating or reversing the main clinical manifestations of systemic envenomation [1,2,6,7]. However, the potential risk of adverse reactions to antivenom of equine origin and, to a lesser extent, the cost of antivenom production and its general availability, have, in recent decades, led to the investigation of alternative approaches for treating envenomation by *C. d. terrificus*, including the use of natural (plant) products and, more recently, drug repurposing with compounds such as varespladib, a selective PLA_2_ inhibitor [10].

Various natural products (crude extracts and purified compounds), as well as synthetic molecules, have been screened for their ability to attenuate the local and systemic effects of envenomation by *C. d. terrificus* in experimental animals (summarized in Table 1). Many of these studies have investigated the actions of these substances on venom enzymatic activities (mainly phospholipases A_2_-PLA_2_ and metalloproteinases) and biological activities such as edema formation, coagulant and hemolytic activities, renal dysfunction, neuromuscular blockade in vitro, and lethality.

In previous work, we examined the ability of several plant extracts and purified compounds to neutralize or reverse the neuromuscular blockade caused by *C. d. terrificus* venom in vitro. These investigations identified theoflavin, but not epigallocatechin gallate (both from *Camellia sinensis*), as a molecule of potential interest [21]. Similarly, tannic acid, which is abundant in *Plathymenia reticulata* [32,33], but not *Mikania laevigata* [26,34,35], also protects against this neurotoxicity in vitro [26].

**Figure 1:**
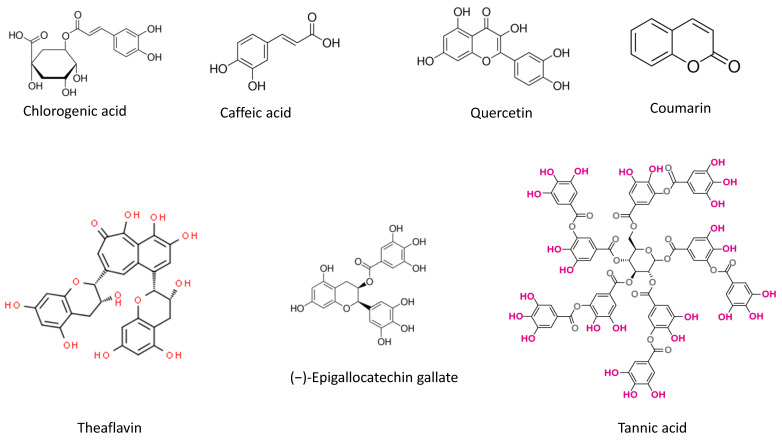
Chemical structures of phytochemicals that have previously been examined for their ability to protect against *C. d. terrificus* venom-induced neuromuscular blockade in mouse isolated phrenic nerve-diaphragm preparations in vitro (coumarin, epigallocatechin gallate, theaflavin and tannic acid) or that were studied in this work (caffeic acid, chlorogenic acid, and quercetin). The presence of theaflavin and tannic acid in *Camellia sinensis* [21] and *Plathymenia reticulata* [26] extracts, respectively, accounts for the ability of these extracts to protect against neuromuscular blockade when compared to extracts rich in coumarin, such as that of *Mikania laevigata,* that do not offer such protection [26]. Hydroxyl groups are highlighted in red and pink in theaflavin and tannic acid. Chlorogenic acid, caffeic acid, and quercetin were investigated in the present study.

In this study, we have extended our investigation of plant compounds as ‘antivenoms’ to examine the ability of caffeic acid, chlorogenic acid, and quercetin (Figure 1) to protect against the neuromuscular blockade caused by *C. d. terrificus* venom in mouse isolated phrenic nerve-diaphragm (PND) preparations. Some of these compounds, e.g., chlorogenic acid [17], are known to attenuate the PLA_2_ activity and inflammation caused by *C. d. terrificus* venom. In addition, we examined the efficacy of intraperitoneally injected tannic acid in protecting against venom-induced AKI and lethality in rats, since this compound is already known to protect against venom-induced neurotoxicity in vitro [26].

## 2. Results and Discussion

### 2.1. Caffeic Acid, Chlorogenic Acid, and Quercetin Do Not Protect against C. d. terrificus Venom-Induced Neuromuscular Blockade In Vitro

The mouse isolated PND preparation is a robust experimental model for testing peripheral skeletal muscle neuromuscular blockade and assessing the neutralizing capacity of antivenoms against the neurotoxicity of a wide variety of venoms [36]. The preincubation of venom or toxin with the compound, extract, or mixture of interest is a standard procedure in toxinological assays and allows rapid assessment of the ability of pharmacological agents to attenuate neuromuscular blockade and thus predict the ‘antivenom’ potential of the compound [37,38,39].

**Figure 2:**
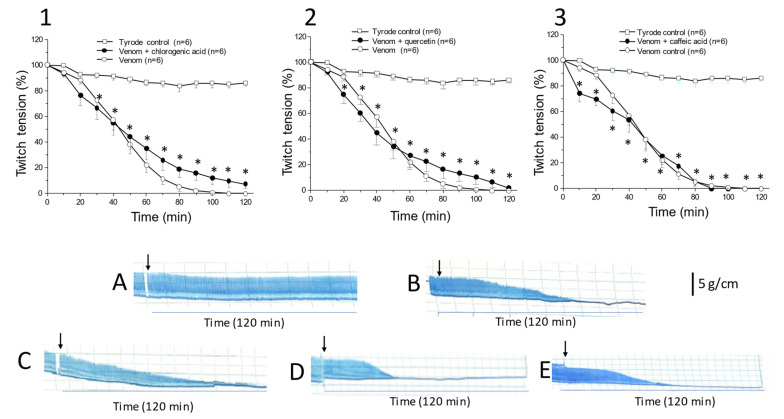
Twitch-tension responses of mouse PND preparations incubated with Tyrode solution (control) and *C. d. terrificus* venom (10 μg/mL) in the absence and presence of chlorogenic acid (1 mg/mL) (**1**), quercetin (0.5 mg/mL) (**2**), and caffeic acid (0.5 mg/mL) (**3**). Panels (**A**–**E**) show representative recordings of the responses to Tyrode (**A**), *C. d. terrificus* venom (**B**), venom + chlorogenic acid (**C**), venom + quercetin (**D**), and venom + caffeic acid (**E**). None of the compounds tested protected against the venom-induced neuromuscular blockade. The preparations were obtained from euthanized mice and mounted for indirect stimulation (supramaximal stimuli, frequency: 0.06 Hz, duration: 0.2 ms) in Tyrode solution under a resting tension of 5 g/cm, as described in the Methods section. The ability of caffeic acid, chlorogenic acid, and quercetin to prevent the venom-induced neuromuscular blockade was assessed by preincubating the venom with each compound separately for 30 min at 37 °C before evaluating the residual activity. The points in graphs **1**–**3** represent the mean ± S.E.M. of the number of experiments indicated. * *p* < 0.05 compared to Tyrode control (the asterisk applies to both curves in each graph).

Figure 2 shows the results of venom + phytochemical preincubations and their respective myographical recordings. The venom concentration tested (10 μg/mL) produced significant neuromuscular blockade in mouse PND preparations starting 20–30 min after addition when compared to control preparations incubated with Tyrode solution alone. Preincubation of venom with single concentrations of caffeic acid (0.5 mg/mL), chlorogenic acid (1 mg/mL), or quercetin (0.5 mg/mL) had no effect on the venom-induced neuromuscular blockade, indicating that these compounds were ineffective in preventing this blockade.

These findings contrast with previous work showing that theaflavin from *C. sinensis* [21] and tannins from *P. reticulata* [26] protect against the paralysis and myotoxicity of *C. d. terrificus* venom in vitro. The protective action of the latter two compounds may involve different mechanisms since theaflavin failed to form precipitates with venom proteins during preincubation, whereas tannic acid formed precipitates [26], as also reported by others [39].

### 2.2. Systemic Effects

Tea polyphenols have been shown to attenuate the local tissue damage induced by *Naja naja kaouthia* Lesson (Elapidae) and *Calloselasma rhodostoma* Kuhl (Viperidae) venoms [40]. Tannic acid alone or in plant extracts also protects against the deleterious effects of Habu snake (*Protobothrops flavoviridis*) and Erabu sea snake (*Laticauda semifasciata*) venoms [41] and attenuates the damage caused by the venoms of *Naja kaouthia* [42,43], *Bothrops jararacussu* [32] and *Bothrops atrox* [44], in addition to protecting against a variety of snake venom and bacterial toxins [45].

Based on previous work, in which we demonstrated a protective effect of tannic acid on the in vitro neuromuscular blockade caused by *C. d. terrificus* venom [26], in this present study, we extended our investigation of tannic acid to examine its possible effects on the manifestations of systemic envenomation by *C. d. terrificus* venom. The effects of caffeic acid, chlorogenic acid, and quercetin were not examined in vivo, as these compounds were ineffective in preventing the venom-induced neuromuscular blockade in vitro (see previous section and Figure 2).

The blood urea level (used as an indicator of the severity of envenomation and development of AKI) increased from 46.7 ± 3.1 mg dL^−1^ in control rats (G1) to 93.7 ± 4.8 mg dL^−1^ with venom alone (G2-venom, 6 mg kg^−1^, i.p.; *p* < 0.05). The preincubation of venom with tannic acid (50 mg; preincubation for 1 h at 37 °C) significantly attenuated this increase in blood urea to 52.0 ± 4.0 mg dL^−1^ (G3-venom preincubated with tannic acid, *p* < 0.05; *n* = 5 each), but no protection was observed when tannic acid was administered 2 h after the venom (G4-venom + tannic acid 2 h later, 90.9 ± 1.5; *n* = 5). In contrast, antivenom (positive control) significantly blunted the increase in blood urea when given 2 h after the venom (G5-venom + antivenom 2 h later, 48.7 ± 4.3, *p* < 0.05; *n* = 5). The venom-induced increase in blood creatinine (from 0.41 ± 0.1 mg dL^−1^ in G1 to 1.3 ± 0.14 mg dL^−1^ in G2, *p* < 0.05; *n* = 5) was attenuated to 0.42 ± 0.04 mg dL^−1^ by preincubation with tannic acid (G3, *p* < 0.05; *n* = 5) but was unaffected by tannic acid (G4: 1.1 ± 0.4 mg dL^−1^, *p* > 0.05; *n* = 5) or antivenom (G5: 1.0 ± 0.07 mg dL^−1^, *p* > 0.05; *n* = 5) given 2 h after venom administration. These protocols showed that tannic acid neutralizes the venom-induced increase in blood urea and creatinine but only when preincubated with the venom; administration after envenomation had no effect on these parameters.

We also examined the effect of tannic acid on venom lethality, assessed as the survival time after venom administration. All control rats (G1-sham group that received only anesthetic) survived for 24 h (the pre-established time limit for assessing survival), whereas rats treated with venom alone (G2-6 mg kg^−1^, i.p.) died after 8.2 ± 0.8 h (*n* = 5). The preincubation of venom with tannic acid (G3-1 h at 37 °C) completely abolished the lethality of the venom, with all rats surviving for 24 h post-venom. Whereas tannic acid given 2 h post-venom had no effect on the blood creatinine and urea concentrations (as indicated above), this compound nevertheless attenuated the lethality of the venom (but did not prevent death), since rats that received tannic acid 2 h after venom (G4) survived for 18.5 ± 3.5 h (*n* = 5), with no additional support measures being used. Rats that received antivenom 2 h post-venom (G5, *n* = 5) also survived for 24 h after envenomation. These findings indicate that the preincubation protocol completely neutralized the lethality of the venom (and was also effective in attenuating the venom-induced increases in blood creatinine and urea) and but was only partially effective in neutralizing the lethality when given 2 h post-venom (whereas blood creatinine and urea were unaffected by this post-venom treatment). Tannic acid was not as effective as antivenom when given 2 h post-venom.

### 2.3. Analysis of the Tannic Acid–Venom Interaction

**Figure 3:**
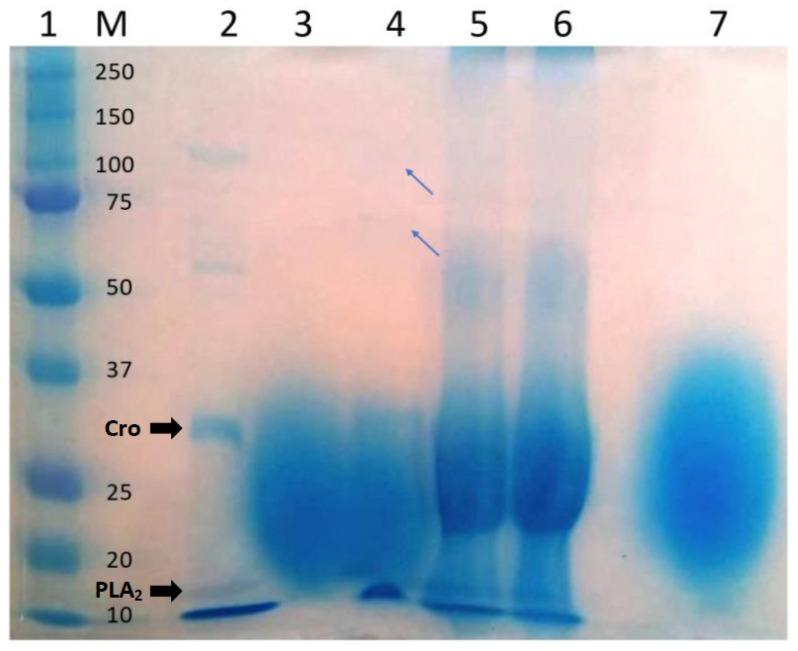
SDS-PAGE electrophoretic profiles of *C. d. terrificus* venom (24 μg; lane 2), supernatant of the venom + tannic acid mixture (10 μL from a mixture of 24 μg venom + 500 μg tannic acid; lane 3), precipitate of the venom + tannic acid mixture (10 μL from a mixture of 24 μg venom + 500 μg tannic acid; lane 4), antivenom–venom mixture (10 μL containing ~10 μg venom + antivenom; lane 5), antivenom (10 μL; lane 6) and tannic acid (500 μg; lane 7). The samples were run on a 10% polyacrylamide gel in the presence of 0.5% (*v/v*) ß-mercaptoethanol and stained with Coomassie Brilliant Blue G-250. Lane 1 contains molecular mass markers (M, kDa). Note the absence of venom proteins in lane 3 (suggesting their removal by interaction with tannic acid) and faint bands of some venom proteins in lane 4 (arrows), possibly released from tannic acid during resuspension of the precipitate—compare with the profile for tannic acid alone (lane 7). The electrophoretic profiles of the antivenom–venom mixture and antivenom alone are shown for comparison. Cro (~30 kDa)—crotoxin (complex of PLA_2_ and crotapotin; principal toxin of the venom), PLA_2_ (~14–15 kDa)—phospholipase A_2_.

Since tannic acid has previously been shown to form precipitates with *B. jararacussu* and *C. d. terrificus* venom proteins [26], we examined whether such interaction could account for the neutralization of lethality seen in the preincubation experiments. For this, the supernatant and precipitate of the preincubated mixture of venom + tannic acid were analyzed by SDS-PAGE. Figure 3 shows the electrophoretic profiles of different samples (venom alone, supernatant and precipitate of the venom + tannic acid mixture, supernatant of the antivenom–venom mixture, and antivenom and tannic acid alone). As shown in lane 3 of Figure 3, the supernatant of the venom + tannic acid mixture (G3) was devoid of venom proteins (based on comparison with venom alone, lane 2), probably because these had been removed by interaction with tannic acid. In support of this conclusion, lane 4 of Figure 3 shows that faint bands corresponding to venom proteins were present in the precipitate of the venom + tannic acid mixture. Studies with other snake venoms, e.g., *Naja kaouthia* [42], have also detected venom proteins in the precipitates of venom + tannic acid mixtures. No bands corresponding to venom proteins (such as seen in lane 2 with venom alone) were detected in the supernatant of the antivenom–venom mixture (lane 5). This may reflect a combination of the removal of venom proteins by interaction with antibodies and the elevated protein concentration of antivenom that perhaps masked the bands corresponding to venom proteins.

Although preincubation protocols prior to testing are useful for initial assessment of the potential neutralizing capacity of antivenoms or other compounds of interest [37,38], this approach does not reflect the clinical situation in which treatments are given after the envenomation. In addition, at least in the case of tannic acid, the precipitation of blood proteins after i.v. injection of this compound makes its administration by this route unfeasible, but this problem may be circumvented by using other routes of administration (intraperitoneal—i.p.; subcutaneous—s.c.). Intraperitoneal injection was used by Yugarani et al. [46] to administer tannic acid dissolved in normal saline, twice a week at a dose of 15 mg/rat/injection (30 mg/week). The dose used by these authors was lower than that used in our study (50 mg), but the period of exposure here (24 h) was much shorter than that used by Yuragani et al. [46] (10 weeks); there were no adverse responses to the treatment in the latter study. Xue et al. [47] treated rats with 40 mg of tannic acid/kg/day for seven days and observed good tolerance to this compound. Drugs administered into the peritoneal cavity diffuse into the surrounding tissues, where they may be metabolized by or bound to tissue proteins or be absorbed by the microcirculation or lymph vessels to reach the general circulation [48].

### 2.4. Other Parameters Analyzed

**Figure 4:**
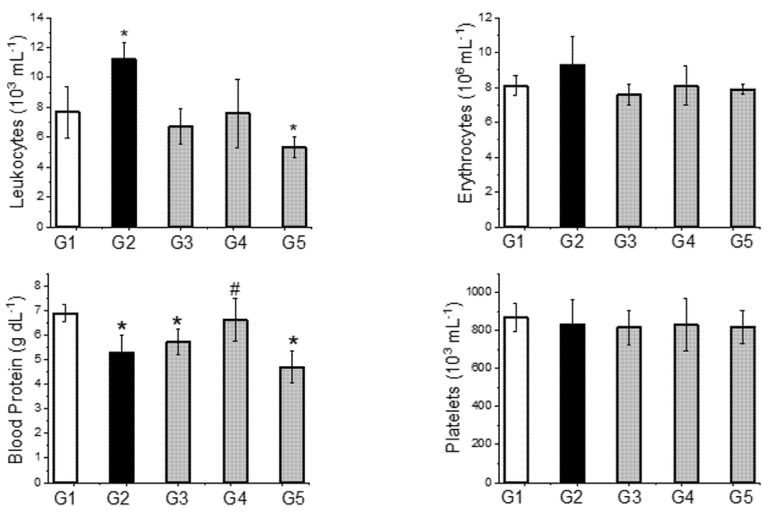
Blood cell counts (leukocytes, erythrocytes, and platelets) and blood protein concentrations in rats injected with *C. d. terrificus* venom (6 mg kg^−1^, i.p.) and subjected to different treatments. G1—control (sham) rats, G2—venom alone, G3—supernatant from a preincubated mixture (1 h at 37 °C) of venom (2.4 mg for a 400 g rat) + tannic acid (50 mg), G4—tannic acid (50 mg) administered 2 h post-venom, and G5—antivenom (4 mg kg^−1^, i.p., corresponding to 1.6 mL for a 400 g rat) administered 2 h post-venom. Note the venom-induced leukocytosis that was normalized by the other treatments (antivenom caused a slight reduction in leukocyte number); the venom had no effect on erythrocyte and platelet numbers. There was a significant reduction in blood proteins that was normalized by tannic acid given 2 h post-venom, but not by the other treatments. The columns represent the mean ± SEM (*n* = 5). * *p* < 0.05 compared to sham rats (G1). ^#^ *p* < 0.05 compared to venom alone (G2).

Figure 4 shows the effect of tannic acid and antivenom on the blood cell counts (leukocytes, erythrocytes, and platelets) and blood protein concentrations in the different experimental groups.

Although the degree of hydration can influence blood protein concentrations [49], the changes observed here may be indicative of renal damage that is reflected in proteinuria [50]. Indeed, proteinuria is considered a marker of early vascular disease and abnormal endothelial function (including in the glomerular microvasculature) and is indicative of the severity and duration of other risk factors (e.g., elevated blood pressure) rather than playing a pathogenic role per se [51,52]. Proteinuria is well-known in envenomation by *C. d. terrificus* [1,6] and, in agreement with this, proteinuria was observed after venom injection (proteinuria, mg dL^−1^, *n* = 5 each: G1-Sham 136.2 ± 20.0 vs. G2-Venom 373.6 ± 155.5; *p* < 0.05) and was attenuated by preincubation with tannic acid (G3 69.5 ± 21.6 mg dL^−1^; *p* < 0.05 compared to G1 and G2) and by the administration of tannic acid (G4 129.2 ± 18.3 mg dL^−1^; *p* < 0.05 compared to G2) or antivenom (G5 180.2 ± 24.3 mg dL^−1^; *p* < 0.05 compared to G1 and G2) 2 h post-venom. Preincubation with tannic acid was more effective in attenuating proteinuria than when this compound was given 2 h post-venom. There was no significant difference between the protection offered by tannic acid and antivenom when both were given 2 h post-venom. Treatment with tannic acid 2 h post-venom appeared to provide the best overall protection since this was the only intervention that normalized the blood and urinary protein levels together.

**Figure 5:**
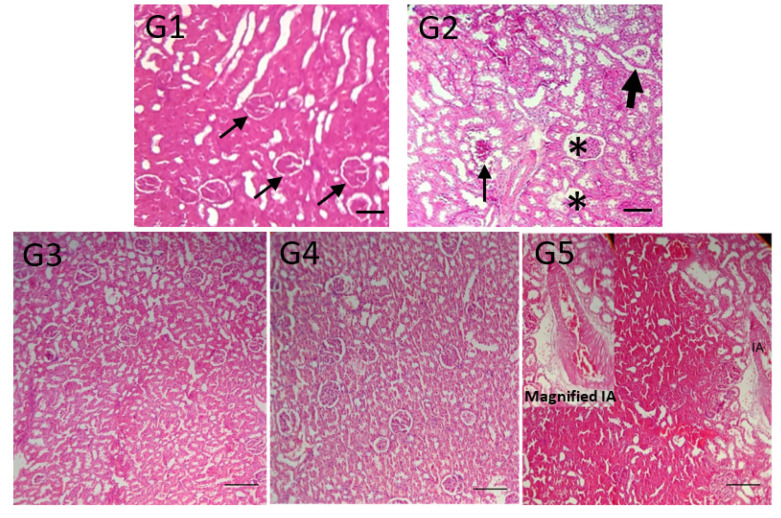
Histological appearance of renal cortex from a control rat (**G1**), *C. d. terrificus* venom-treated (6 mg kg^−1^, i.p.) rat (**G2**), and rats injected with supernatant from a preincubated mixture of venom + tannic acid (**G3**) or with tannic acid (**G4**) or antivenom (**G5**) 2 h after venom injection. The inset in panel (**G5**) shows a magnified interlobular arteriole (IA). (**G1**)—Section from a control rat showing the normal appearance of the tissue architecture, proximal and distal tubules, and several renal corpuscles (arrows). (**G2**)—Section from a venom-treated rat showing changes in the renal corpuscles and Bowman’s capsule (*) indicative of glomerular lesions, with ischemic acute tubular injury (small arrow), hemorrhagic content and tubular dilation (large arrow). Overall, the three treatments (**G3**–**G5**) preserved most of the renal corpuscles. See Figure 4 legend for further experimental details (amounts and doses/concentrations used, incubation conditions, etc.). The tissue sample in group (**G3**) was collected 24 h after injection of the supernatant, while those in groups (**G4**,**G5**) were collected ~18 h and 24 h after venom injection. The tissue sections were stained with hematoxylin-eosin. Scale bar = 20 µm.

Figure 5 shows the histological appearance of renal tissue from sham rats (G1, control) and rats injected with venom alone (G2). Rats in the latter group had glomerular lesions and interstitial injury that could explain the proteinuria indicated above [50]. Despite the differences in proteinuria noted above, all treatments (G3, G4, and G5) attenuated the venom-induced lesions in renal tissue.

## 3. Further Considerations and Conclusions

We have previously shown that theoflavin [21] and tannic acid [26] are effective in protecting against snake venom-induced neuromuscular blockade in vitro. Of the three additional compounds studied here (caffeic acid, chlorogenic acid, and quercetin), quercetin has been the most studied in relation to its effects on snake venoms. The findings of this investigation indicate that none of these compounds was effective in protecting against the in vitro neuromuscular paralysis caused by *C. d. terrificus* venom.

The lack of protection by caffeic acid agreed with a similar lack of effect against the blockade caused by PrTX-I, a Lys49-PLA_2_ from *Bothrops pirajai* venom in mouse PND preparations, despite protecting against toxin-induced myonecrosis in diaphragm muscle; indeed, the highest PrTX-I:caffeic acid ratio tested enhanced the speed of blockade without markedly affecting the maximum blockade achieved [53]. The lack of effect on neuromuscular blockade by caffeic acid contrasts with that of rosmarinic acid, a derivative of caffeic acid, that markedly attenuates the neuromuscular blockade by PrTX-I [54]. The difference between the activities of these two molecules is attributed to their distinct binding sites on the PLA_2_ molecule [53].

Although chlorogenic acid has been reported to interact with PLA_2_ from *Dabioa russelii* (Russell’s viper) venom [55], and a hydroalcoholic extract of *Vellozia flavicans* Mart. ex. Schult. containing chlorogenic acid, quercetin, and other compounds has been shown to protect against the neuromuscular blockade by *Bothrops jararacussu* venom in mouse PND preparations in vitro [56], the effect of chlorogenic acid alone on snake venom neurotoxicity has not previously been assessed. Our findings indicate that chlorogenic acid is ineffective in protecting against neuromuscular blockade in vitro, at least against that produced by *C. d. terrificus* venom.

Quercetin and derivatives are well known for their ability to interact with snake venom PLA_2_ [57,58,59,60]. Quercetin interacts with *C. d. terrificus* PLA_2_ to inhibit enzymatic activity and platelet aggregation but has little or no effect on mouse paw edema [20,59], with a glycated form of quercetin (3-rhamnosylquercetin) being more effective at protecting against the PLA_2_-induced edema and myotoxicity [59]. Quercetin also protects against the cytotoxicity of *C. d. terrificus* venom in mouse hippocampal slices [60]. The effect of quercetin on venom-induced neuromuscular blockade varies. Quercetin (10 μg/mL) protected against the marked muscle facilitation caused by crotamine (a low molecular mass ~4 kDa, basic myotoxin from *C. d. terrificus* venom) and against the neuromuscular blockade caused by crotoxin in mouse PND preparations [60]. In contrast, as shown here, quercetin did not protect against neuromuscular blockade, even at a high concentration (0.5 mg/mL); the reason for this discrepancy is unclear. In chick biventer cervicis nerve-muscle preparations, quercetin slightly delayed the onset and progress of neuromuscular blockade but did not prevent the final blockade [20].

Tannins are common in plant extracts investigated for anti-venom activity, and various studies have examined the effects of purified tannins on snake venoms [41,42,43,44,45,61,62] and their toxins, especially PLA_2_ [15,63]. Kupussamy and Das [61] investigated the protective effect of tannic acid injected subcutaneously (s.c.) on the lethality and other actions of *Crotalus admanteus* (eastern diamonback rattlesnake) venom in mice. When co-injected with five lethal doses (LD_50_) of venom, tannic acid (30 mg/kg) provided much greater protection against lethality than the flavonoids apigenin, kaempferol, and luteolin. Total protection was observed when the venom (2 LD_50_) was preincubated with tannic acid, less protection was observed with simultaneous injection, and the least protection was observed when tannic acid was given 5–10 min after the venom. Our findings with *C. d. terrificus* venom were similar to these, i.e., tannic acid protected completely against lethality when preincubated with the venom prior to administration, whereas administration 2 h post-venom delayed the time of death but did not reduce the lethality.

Envenomation by *C. adamanteus* can cause extensive myonecrosis, and Kuppusamy and Das [61] noted that preincubation of venom with tannic acid virtually abolished the increase in plasma creatine kinase (a marker enzyme for skeletal myonecrosis), whereas there was only partial attenuation of this increase when tannic acid was given within 5 min of the venom. Renal malondialdehyde formation (an indicator of oxidative stress) was partially attenuated by the preincubation protocol, whereas there was virtually no protection when tannic acid was given s.c. within 5 min of the venom. As shown here, tannic acid had a variable effect on renal parameters, depending on the protocol used: both preincubation and delayed administration abolished the proteinuria, whereas only the preincubation protocol reduced the increase in blood urea and creatinine. Histological analysis showed that the preincubation and delayed administration protocols attenuated the venom-induced renal damage.

Several aspects of this investigation deserve consideration in future studies. First, the mechanism by which tannic acid interacts with *C. d. terrificus* toxins, e.g., by forming non-specific complexes that may or may not be soluble, or by specific interactions to inhibit/inactivate the toxins, deserves further investigation. The protocols used here did not allow a clear distinction between these possibilities. In this regard, Rodrigues et al. [15] have shown that casuarictin, an ellagitannin isolated from the white mangrove (*Laguncularia racemosa*) forms a 1:1 molar complex with *C. d. terrificus* PLA_2_ without precipitating out of solution; this solubility meant that the effect of this compound could be studied on a variety of biological activities mediated by the PLA_2_. Since these authors did not study intact crotoxin (PLA_2_ + crotapotin, such as occurs in the venom), it is unclear whether interaction with this ellagitannin would be different (both in molar ratio and solubility). Second, the most appropriate route of administration needs to be determined. While i.p. administration may be useful and simple in experimental animals, it is not used clinically for the treatment of snakebites (antivenom administration is normally i.v.). Subcutaneous administration is easier than i.p. and has been used in experimental envenomation [61]. Associated with this point is the need to determine the appropriate dose range and develop a suitable formulation of tannic acid that could allow administration by more than one route. Third, the optimal post-venom interval for tannic acid administration needs to be established and would benefit from a pharmacokinetic analysis of tannic acid absorption and distribution from the site of application. Such an analysis is necessary in view of the considerable time lag that can occur between bites by *C. d. terrificus* and admission to hospital for treatment [6]. A short window of opportunity for tannic acid application after envenomation may considerably limit the potential usefulness of this compound.

In conclusion, the results of this investigation have shown that caffeic acid, chlorogenic acid, and quercetin were ineffective in attenuating the neuromuscular blockade caused by *C. d. terrificus* venom in vitro. On the other hand, tannic acid is a more promising molecule for use in the treatment of snakebite, since, in addition to its ability to protect against neuromuscular blockade in vitro [26], our results show that it also provides protection in vivo and could be a potentially useful compound for treating envenomation by *C. d. terrificus*. This conclusion is supported by previous suggestions that tannic acid could be a lead molecule for the development of novel therapeutics for the treatment of snakebites [15,61].

## 4. Material and Methods

### 4.1. Phytochemicals

All phytochemicals (caffeic acid, chlorogenic acid, quercetin, and tannic acid) were purchased from Sigma-Aldrich (Munich, Germany).

### 4.2. Venom

*Crotalus d. terrificus* venom was collected manually from 20 male and female adult snakes (200–400 g, ~2 years-old), captured in the Paraiba Valley close to the city of São José dos Campos (23°11′0″ S; 45°53′0″ W), São Paulo state, and housed in the serpentarium of the Center for Nature Studies at the University of Vale do Paraiba (UNIVAP, São José dos Campos, SP, Brazil) under permit No. SMA 15.380/2012, issued by the São Paulo state Environmental Secretary. The use of this venom was registered with the Brazilian National System for the Management of Genetic Patrimony and Associated Traditional Knowledge (SISGEN, registration No. ACB5FCO/2013). The venom was lyophilized and stored at 4–8 °C until used. All venom quantities mentioned in this work refer to dry weight.

### 4.3. Antivenom

Therapeutic crotalic antivenom raised against *C. d. terrificus* venom (lot 135202/I, expiry date for human use: October 2016) produced by the Instituto Vital Brazil (Rio de Janeiro, RJ, Brazil), was kindly donated by the Escritório Regional de Saúde (ERSA), Piracicaba city, SP, Brazil. The antivenom was used in in vivo experiments in rats and in SDS-PAGE. The amount of antivenom was calculated according to the manufacturer’s stated potency, in which 1 mL of antivenom neutralizes the lethal activity of 1.5 mg of reference *C. durissus* ssp. venom in mice.

### 4.4. SDS-Polyacrylamide Gel Electrophoresis (SDS-PAGE)

The ability of tannic acid (50 mg, the same amount used in the in vivo experiments) to interact with venom proteins was assessed using an in vitro simulation in which venom alone (2.4 mg), the supernatant and precipitate of a mixture of venom preincubated with tannic acid, the supernatant of a mixture of venom preincubated with antivenom, antivenom alone, tannic acid alone, and 10 µL of molecular mass markers (Precision Plus Protein ™ Dual Color, Bio-Rad, San Diego, CA, USA) were run on a 10% polyacrylamide gel in sodium dodecyl sulfate-polyacrylamide gel electrophoresis (SDS-PAGE). The samples were prepared in 2× standard buffer (65.8 mM Tris-HCl, 26.3% glycerol, 2.1% SDS and 0.01% bromophenol blue) containing 0.5% β-mercaptoethanol (Bio-Rad) at 100 °C for 10 min. The gel was run in a vertical Mini-Protean electrophoresis system (150 V, 30 mA, 15 W, 45 min), stained for 30 min with Coomassie Brilliant Blue G-250 (Bio-Rad) and washed in deionized water, essentially as described elsewhere [8,64]. The amount of venom to be used was calculated based on a rat weighing 400 g. Thus, 2.4 mg of venom or 50 mg of tannic acid were each dissolved in 1 mL of phosphate-buffered saline (PBS). The volumes were mixed and incubated for 1 h at 37 °C, followed by centrifugation. Aliquots of 10 µL each were loaded into each well of the gel.

### 4.5. Animals

Male Wistar rats (*Rattus norvegicus*) (290–400 g; 3–6 months old) and male Swiss mice (20– 30 g; 1 month old) were purchased from the Central Animal Facility of the Instituto de Ciências Biomédicas, Universidade de São Paulo, SP, Brazil, and housed (3 rats/plastic cage or 4 mice/plastic cage on a wood shavings substrate) at the University of Sorocaba animal facility at 22 ± 3 °C and 50 ± 5% humidity on a 12 h light/dark cycle (lights on at 6 a.m.), with access to food and water ad libitum.

Standard laboratory practices for animal care were followed according to the Guide for the Care and Use of Laboratory Animals [65] and the Animal Research: Reporting of In vivo Experiments (ARRIVE) guidelines [66]. The experimental protocols were approved by the institutional Committee for the Care and Use of Experimental Animals at the University of Sorocaba (protocol No. 031/2016, extended until 2020).

### 4.6. Mouse Phrenic Nerve-Diaphragm Preparations

Mouse phrenic nerve-diaphragm (PND) preparations were isolated as described elsewhere [67,68,69] and carefully mounted under a tension of 5 g/cm in a 5 mL organ bath containing Tyrode solution (composition, in mM): NaCl 137, KCl 2.7, CaCl_2_ 1.8, MgCl_2_ 0.49, NaH_2_PO_4_ 0.42, NaHCO_3_ 11.9, and glucose 11.1, pH 7.4, aerated with 95% O_2_ and 5% CO_2_. The preparations were indirectly stimulated through the phrenic nerve with supramaximal stimuli at a frequency of 0.06 Hz and duration of 0.2 ms, using an ESF-15D double physiological stimulator. Isometric twitch tension was recorded with a force-displacement transducer (cat. No. 7003, Ugo Basile, Italy) coupled to a digital recorder system (Data Capsule, cat. No. 17400, Ugo Basile) containing a Basic Preamplifier (cat. No. 7080, Ugo Basile), coupled to a computer via a USB interface for data storage.

The preparations were allowed to stabilize for at least 20 min before starting the experiments. After recording twitch tension responses for 10 min under basal conditions, the pharmacological protocols (*n* = 6 per protocol) were initiated by adding *C. d. terrificus* venom (10 µg/mL) alone or after preincubation (30 min, 37 °C) with caffeic acid (0.5 mg/mL), chlorogenic acid (1 mg/mL), or quercetin (0.5 mg/mL). The concentrations of the phytochemicals were chosen based on previous assays that showed no interference on the basal twitch-tension responses of the neuromuscular preparations (data not shown).

### 4.7. In Vivo Experiments

#### 4.7.1. Selection of *C. d. terrificus* Venom Dose for Severe Envenomation

A venom dose of 6 mg kg^−1^, i.p., was used to induce severe envenomation based on the prior determination of blood urea (before and after venom: 46.7 ± 3.1 and 93.7 ± 4.8 mg dL^−1^, respectively; *p* < 0.05) and creatinine (before and after venom: 0.41± 0.1 and 1.3 ± 0.1 mg dL^−1^, respectively; *p* < 0.05) concentrations using commercial kits, according to the manufacturer’s recommendations (Labtest^®^, Lagoa Santa, MG, Brazil). Renal damage was confirmed by histological analysis, as described elsewhere [70].

#### 4.7.2. The Efficacy of Tannic Acid against the Lethality of *C. d. terrificus* Venom

**Figure 6:**
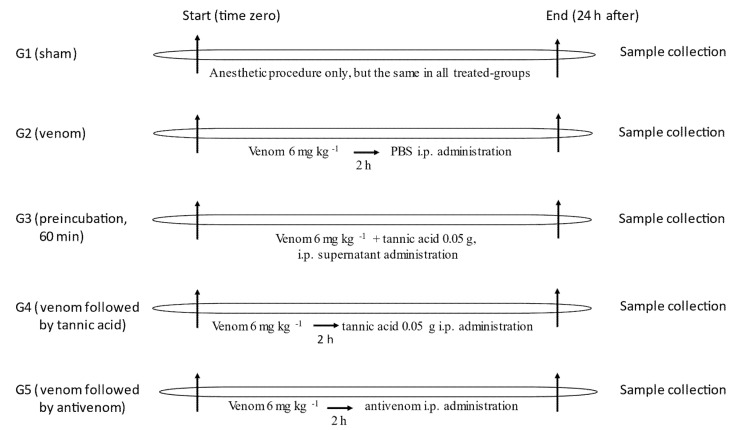
Experimental design for the in vivo study (*n* = 5, each). Groups (**G3**–**G5**) were compared with groups (**G1**,**G2)** with regard to lethality, blood biochemical and hematological parameters, and renal histological alterations.

Since tannic acid has previously been shown to protect against the neurotoxicity of *C. d. terrificus* venom in vitro [26], we examined the ability of this phytochemical to protect against the effects of this venom in vivo. Rats were allocated to five experimental groups: (1) Group 1 (G1)-Sham (*n* = 5): rats that received only anesthetic, since all groups received anesthesia + treatment. The anesthetic mixture consisted of xylazine (10 mg kg^−1^) and ketamine (10 mg kg^−1^) (both purchased from Ceva^®^, Paulínia, SP, Brazil), with midazolam (1 mg kg^−1^) being used for initial sedation (before venom administration) and tramadol (5 mg kg^−1^) as an analgesic (both from Medley^®^, Campinas, SP, Brazil) after venom administration, (2) Group 2 (G2)-*C. d. terrificus* venom (6 mg kg^−1^ body weight, i.p.; *n* = 5): rats were injected with venom alone, (3) Group 3 (G3)-venom (6 mg kg^−1^) + tannic acid (50 mg) (*n* = 5): rats were injected with venom preincubated with tannic acid for 1 h at 37 °C. After preincubation, the mixture was centrifuged and the supernatant was injected i.p., (4) Group 4 (G4)-venom followed 2 h later by tannic acid (*n* = 5): rats were injected with venom, and 2 h later, tannic acid (50 mg) was injected i.p., (5) Group 5 (G5)-venom followed 2 h later by antivenom (*n* = 5): rats were injected with venom, and 2 h later, antivenom (4 mL kg^−1^) was injected i.p. For all groups, the effect of the treatment on venom lethality was assessed by recording the survival time. Rats that survived for 24 h (time limit for monitoring survival) were euthanized using isoflurane (Cristália^®^, Itapira, SP, Brazil). Figure 6 summarizes the experimental groups and protocol.

#### 4.7.3. Analytical Procedures Performed on Blood and Renal Tissue Samples

Additional parameters analyzed in blood and/or renal samples in each group included hematological parameters (leukocyte, erythrocyte, and platelet counts), blood protein concentrations, and proteinuria, in addition to histological alterations. The hematological parameters were analyzed in triplicate using a Sysmex XS 1000i™ Hematology Analyzer (Roche, Basel, Switzerland) [71].

### 4.8. Data Analysis

All numerical data were expressed as the mean ± SEM. The normality of the data was assessed using the Shapiro–Wilk test. Statistical comparisons between two groups were done using Student’s *t*-test, whereas comparisons involving ≥3 groups were done using one-way ANOVA followed by the Tukey multiple test, with *p* < 0.05 indicating significance in all cases. All data analyses were done using Origin^©^ v.8.0 (OriginLab Corporation, Northampton, MA, USA).

## Figures and Tables

**Table 1:** Compounds screened for protection against the local and systemic effects of *C. d. terrificus* venom in experimental animals.

Substance(s)	Activity Evaluated	Main Findings	Reference
Varespladib (LY315920) and its orally bioavailable prodrug, methyl-varespladib (LY333013)	Protection against the lethality of neurotoxic snake venoms (*Notechis scutatus*, *Crotalus durissus terrificus*, *Bungarus multicinctus, Oxyuranus scutellatus*) in mice.	Varespladib abrogated or delayed the neurotoxic manifestations induced by some venoms in which neurotoxicity was mainly dependent on presynaptically active PLA_2_s. LY315920 reversed the paralytic manifestations in severely envenomed mice.	[10]
Vanillic acid	Inhibition of PLA_2_ and proteases.	Vanillic acid inhibited the PLA_2_ activity of *Bothrops alternatus* (∼25% inhibition) and the caseinolytic activity of *Bothrops atrox* (∼30%), *Bothrops jararacussu* (∼44%), and *C. d. terrificus* (∼33%).	[11]
Antibodies against synthetic peptides	Antigenicity/immunogenicity of crotoxin and crotamine.	Antibodies against synthetic peptides protected mice against venom lethality.	[12]
Aqueous and methanolic extracts of *Plinia jaboticaba* skins	Inhibition of PLA_2_ and proteases.	Inhibition of the PLA_2_ activity of *Bothrops moojeni* and *Crotalus durissus terrificus* venoms, but not *B. atrox* venom. The greatest inhibition of hemolysis was observed for the methanolic extract when incubated with *B. moojeni* and *C. d. terrificus* venoms (inhibition of 21–100%). Thrombolysis induced by *B. moojeni* and *C. d. terrificus* venoms was inhibited by both extracts (by 32–83% and 51–83% for the aqueous and methanolic extracts, respectively).	[13]
Essential oil from *Lippia origanoides*	Inhibition of PLA_2_ activity.	Potentiation of hemolytic activity in preincubation protocols and presence of prothrombotic activity.	[14]
Casuarictin from *Laguncularia racemosa*	Inhibition of secretory PLA_2_ (sPLA_2_).	The anti-inflammatory activity suggested a potential use of this compound in treating edema and myonecrosis induced by sPLA_2_.	[15]
8-*C*-rhamnosyl apigenin from *Peperomia obtusifolia*	Inhibition of sPLA_2_ and cytosolic PLA_2_ (cPLA_2_).	Inhibition of *C. d. terrificus* sPLA_2_ and cPLA_2_, but also significant inhibition of cyclooxygenase activity.	[16]
Chlorogenic acid (5-caffeoylquinic acid, 5CQA), isolated from *Baccharis oxyodonta*	Effect on sPLA_2_ structure and pharmacological activity.	5CQA modulated the inflammatory activity of sPLA_2_.	[17]
N-acetyl-cysteine	Protection against venom-induced renal damage.	The renal protection observed with NAC suggested a potential usefulness, along with antivenom therapy, in envenomation by *C. d. terrificus*.	[18]
Allopurinol and probenecid	Effects of allopurinol and probenecid on venom-induced renal dysfunction.	Allopurinol deserves to be clinically evaluated as an ancillary treatment for snakebite along with antivenom.	[19]
Quercetin	Inhibition of sPLA_2_.	Quercetin inhibited the enzymatic activity and some pharmacological activities of sPLA_2_, including its antibacterial activity, its ability to induce platelet aggregation, and its myotoxicity, but did not reduce the inflammatory and neurotoxic activities of sPLA_2_.	[20]
*Camellia sinensis* extract and its metabolites theoflavin and epigallocatechin gallate	Inhibition of venom-induced in vitro neuromuscular blockade.	The extract and theoflavin, but not epigallocatechin gallate, protected against irreversible neuromuscular blockade induced by *C. d. terrificus* venom in mouse phrenic-nerve diaphragm.	[21]
Lipoic acid	Effects of lipoic acid (LA) on lethality, renal dysfunction, aminopeptidase and GSSG/GSH levels in venom-injected mice.	LA solubilized/removed proteins from the membrane-bound fraction with impairment of most aminopeptidase activity but could still be useful for the treatment of directly induced venom nephrotoxicity.	[22]
Aqueous extract from *Mikania glomerata*	Inhibition of PLA_2_s, metalloproteinases and serine proteinases.	PLA_2_ activity and *C. d. terrificus* venom-induced edema were inhibited around 100% and ∼40%, respectively. Total inhibition of clotting activity.	[23]
	Attenuation of clinical and laboratory manifestations of venom in Wistar rats.	Envenomation caused hypothermia, local edema, sedation, and a decrease in locomotion. The extract enhanced the recovery from sedation.	[24]
Genetically modified *Eclipta alba* and active coumestans	Inhibition of PLA_2_ and venom-induced myotoxicity.	Clone 19 and isolated coumestans (wedelolactone and demethylwedelolactone) inhibited the myotoxic activity of venom PLA_2_.	[25]
Tannic acid	Inhibition of venom-induced in vitro neuromuscular blockade.	Tannic acid abolished the venom-induced paralysis.	[26]
Aqueous extract of *Schizolobium parahyba* (Caesalpinoideae) leaves	Inhibition of PLA_2_ and biological activities of *C. d. terrificus* venom.	The aqueous extract of *S. parahyba* neutralized PLA_2_ and biological activities (e.g., coagulant activity) of the venom.	[27]
Alkaloid from *Tabernaemontana catharinensis*	Inhibition of venom lethality and myotoxicity.	*Tabernaemontana catharinensis* could be a useful source for model molecules to neutralize the lethality and myotoxicity of *C. d. terrificus* venom.	[28]
Heparin	The effects of crotapotin (a non-toxic and non-enzymatic acid polypeptide naturally complexed with PLA_2_ in the venom) and of heparin on rat paw edema induced by different sPLA_2_. The ability of crotapotin to modulate the enzymatic activity of sPLA_2_ was also evaluated.	Despite the great homology between the various types of sPLA_2_, they interacted with crotapotin on cell surfaces in different ways, leading to either inhibition or potentiation of the paw edema by a mechanism unrelated to their enzymatic activity.	[29]
Encapsulated crotoxin in liposomes	Assessment of immunogenicity.	Crotoxin encapsulated into dehydration-rehydration vesicles (DRV/crotoxin) was less toxic than crotoxin emulsified in Freund’s complete adjuvant (FCA/crotoxin) and induced lower levels of anti-crotoxin antibodies but similar levels of protection when inoculated at high doses (20 or 70 μg of crotoxin/mouse). When DRV/crotoxin was adsorbed to alum at the time of immunization, it induced antibody and protection levels comparable to those produced by FCA/crotoxin.	[30]
Gangliosides	Evaluation of ability of a mixture of gangliosides to neutralize the effects of venom in vitro and in vivo.	Gangliosides effectively neutralized the toxic effects of venom in vitro and in vivo and the intramuscular injection of gangliosides after venom administration protected envenomed animals.	[31]

## Data Availability

All the data relevant to this study have been included in this report. There are no supporting data.
